# Synergistic Stabilization of Nanoemulsion Using Nonionic Surfactants and Salt-Sensitive Cellulose Nanocrystals

**DOI:** 10.3390/polym15244682

**Published:** 2023-12-12

**Authors:** Lingling Liu, Kyle A. E. Abiol, Mason A. Friest, Kaleb D. Fisher

**Affiliations:** 1 Department of Agricultural and Biosystems Engineering, Iowa State University, Ames, IA 50010, USA; 2 Department of Chemical and Biological Engineering, Iowa State University, Ames, IA 50010, USA; 3 Department of Mechanical Engineering, Iowa State University, Ames, IA 50010, USA; 4 Roy J. Carver Department of Biochemistry, Biophysics and Molecular Biology, Iowa State University, Ames, IA 50010, USA

**Keywords:** nanocellulose, salt, nanoemulsion, surfactant, essential oil

## Abstract

Soybean stover is a lignocellulose biomass that is rich in cellulose. In the present study, soybean cellulose nanocrystals (CNCs) were prepared from soybean stover by alkaline treatment, bleaching treatment, acid hydrolysis, dialysis and ultrasonication. The as-prepared soybean CNC was characterized by transmission electron microscopy (TEM), zetasizer and rheometer. The effects of NaCl on the particle size, zeta potential, and viscosity of soybean CNC was studied. Soybean CNC was explored as an emulsion stabilizer for lemongrass-essential-oil-loaded emulsions. Soybean CNCs could stabilize the oil-in-water emulsion against coalescence but not flocculation. The addition of NaCl reduced the creaming index and enhanced the encapsulation efficiency and freeze–thaw stability of the CNC-stabilized emulsion. Salted CNC (i.e., CNC in the presence of NaCl) enhanced the thermodynamic stability (i.e., heating–cooling and freeze–thaw stability) of Tween 80 stabilized emulsion, while unsalted CNC did not. Synergistic effects existed between Tween 80 and salted CNC in stabilizing oil-in-water emulsions. The nanoemulsion stabilized with Tween 80 and salted CNC had a mean particle size of ~70 nm, and it was stable against all thermodynamic stability tests. This is the first study to report the synergistic interaction between salted CNC and small molecular weight surfactants (e.g., Tween 80) to improve the thermodynamic stability of nanoemulsion.

## 1. Introduction

In recent years, there has been growing interest in developing products from natural resources. Cellulose, being one of the most prevalent substances on Earth, has garnered significant interest owing to its renewable nature, safety, biodegradability, and biocompatibility. Nanocellulose refers to cellulosic material at the nanoscale, characterized by at least one dimension measuring less than 100 nm. The subject of nanocellulose has gained considerable attention in recent years, and it has been applied in various fields [1,2]. As a cellulose material with nanoscale size, nanocellulose has outstanding characteristics including a high aspect ratio, large specific surface area, and high viscosity under certain conditions. Nanocrystalline cellulose, also known as cellulose nanocrystals (CNCs), is a type of nanocellulose with high crystallinity. CNCs are typically produced through the sulfuric acid hydrolysis of cellulose materials, resulting in a width ranging from 5 to 70 nm and a length of 100 to 250 nm, or even extending to several microns [3]. Having a cellulose content of approximately 42% [4], soybean stovers are widely available, low-cost sources for the preparation of nanocellulose. So far, cellulose nanofibrils or cellulose nanocrystals have been prepared from soybean straw, soybean residues or soybean hull via mechanical methods [5] and chemical or enzymatic methods [6]. However, information is limited regarding the potential application of soybean nanocellulose, especially in the formulation of emulsion-based products. 

Emulsions can generally be stabilized by a single emulsifier or emulsion stabilizer. However, many commercial emulsion-based products are formulated with multiple emulsifiers or emulsion stabilizers, including surfactants, polymers and particles [7]. Mixed emulsifier systems may exhibit synergistic effects on emulsion properties and may be cheaper than a single emulsifier [7]. Surfactants and polymers can simultaneously concentrate at the oil–water interface, making the emulsion interface layer more compact and enhancing the emulsion stability [8,9]. Synergistic effects for the stabilization of emulsions have been found between polymers and surfactants, such as hydroxypropylmethyl cellulose and sodium dodecylsulfate [8], as well as maltodextrin and Tween 80 [9]. The combination of polymers and surfactants could result in more stable emulsions than those stabilized by either a polymer or surfactant [8,10].

CNCs have been shown to be Pickering emulsion stabilizers [11]. CNCs can adsorb at the oil–water interface, particularly along its less polar crystalline phase. However, they exhibit only intermediate wettability and do not form micelle-like aggregates, raising certain concerns [12]. The hydrophilicity of CNCs led to poor emulsification performance and limited their application [13]. The addition of salt or lowering pH were shown to be effective in increasing the emulsion phase of CNC-stabilized emulsions [14], however, information is limited on the impact of NaCl on the thermodynamic stability of CNC-stabilized Pickering emulsions. In addition, knowledge is lacking regarding the influence of a mixed emulsifier system (NaCl + CNC and surfactants) on emulsion properties. We hypothesized that by incorporating salt and small molecular weight surfactants with CNCs, a stable nanoemulsion could be formulated. This study explored the influence of NaCl on the characteristics of a Pickering emulsion stabilized by CNCs, along with its synergistic impact when combined with a nonionic surfactant (such as Tween 80) on the stabilization of oil-in-water emulsions. In this study, we highlight the synergistic effects of salted CNCs and Tween 80 in enhancing thermodynamic stability of oil-in-water emulsions loaded with lemongrass essential oil. The results of this study open avenues for the prospective use of CNCs in conjunction with other surfactants in the formulation of stable emulsion products. Moreover, essential oil encapsulation finds applications in various domains, encompassing areas such as food, agriculture, cosmetics, medicine, and packaging. 

## 2. Materials and Methods

Soybean stover was obtained from Iowa State University Research Farms (Boone, IA, USA) and reduced to a 1/8″ size using a knife mill. Lemongrass essential oil (*Cymbopogon schoenanthus* Oil, 100%, reagent grade) was obtained from Spectrum Chemical MFG Corp (New Brunswick, NJ). Tween 80 (P1754) (Sigma-Aldrich, Inc. (St. Louis, MO, USA), sulfuric acids (95–98%) (chemPUR, Karlsruhe, Germany), glacial acetic acids (Fisher scientific, Hampton, NH, USA), sodium hydroxide (Macron Fine Chemicals, Center Valley, PA, USA), sodium chlorite (Beantown chemical, Hudson, NH, USA), sodium chloride (VWR International, Radnor, PA, USA) and dialysis tubing (Cellulose, 12–14 kDa MWCO) (Chemical MFG Corp, New Brunswick, NJ, USA) were used in this study. 

### 2.1. Production of Cellulose Nanocrystals from Soybean Stover

Soybean stover was washed, dried and ground with similar procedures as described in our previous publication [15]. The removal of hemicellulose and lignin from soybean stover were performed by using alkaline and bleaching treatment with similar procedures as described in our previous publication [15]. In particular, soybean stover underwent treatment using a 4 wt% sodium hydroxide solution with a ratio of 1 part solid to 20 parts liquid. This process occurred at 95 °C in a water bath (Boekel Scientific, Feasterville-Trevose, PA, USA) with agitation at 120 rpm for a duration of 6 h. The alkaline-treated soybean stover was then washed with distilled (DI) water until the affluent ran clear, followed by drying at 75 °C for 24 h, grinding and passing through a 40-mesh sieve. Following the bleaching process, 3 wt% sodium chlorite and 3 wt% acetic acid were employed with a ratio of 1 part solid to 20 parts liquid. This treatment took place at 80 °C in a water bath with agitation at 80 rpm for 2 h. The bleached sample underwent washing until the effluent pH approached that of DI water. The bleaching treatment was reiterated, followed by drying, grinding, and sieving as previously detailed. The bleached soybean stover then underwent ball milling (Fritsch pulverisette, FRITSCH Milling and Sizing, Inc., Pittsboro, NC, USA) for 10 min at maximum speed to further decrease the particle size. 

The treated soybean stover then underwent sulfuric acid (64 wt%) hydrolysis with a ratio of 1 part solid to 20 parts liquid at 45 °C for 75 min in a water bath with agitation at 120 rpm. The reaction was quenched by adding 10-fold cold water, followed by centrifugation at 13,689× *g* (Sorvall Evolution RC centrifuge, Waltham, MA, USA) for a duration of 10 min at 4 °C. The pellet underwent a repeated washing and centrifugation process, followed by dialysis until the pH of the dialysate closely matched that of water. The suspension after dialysis was then subjected to sonication using a 500 W ultrasonicator (Fisher Scientific, Hampton, NH, USA) for 30 min at 100% amplitude in pulse mode (5 s on/2 s off) in an ice bath. Subsequently, the suspension underwent centrifugation (Eppendorf 5430R, Enfield, CT, USA) at 7745× *g* for a duration of 20 min at 4 °C, followed by collection of the supernatant. The obtained CNC suspension was kept at 4 °C for subsequent utilization. The color of the soybean stover following each treatment was assessed with a colorimeter (3nh, Shenzhen THREENH Technology Co., Ltd., Shenzhen, China). The step yields and overall yields of the soybean stover sample following each treatment were determined using the following equations.

(1) $$Step\;yield(\%)=\frac{Dried\;sample\;weight\;after\;treatment}{Dried\;sample\;weight\;before\;treatment}\times 100$$

(2) $${Overall\;yield}_{n}(\%)={Step\;yield}_{1}\times {Step\;yield}_{2}\ldots \ldots \times {Step\;yield}_{n}$$

where *n* = 1, 2, 3, 4.

### 2.2. Characterization of CNC

The as-prepared soybean CNC suspension was imaged utilizing a JEM-2100 scanning/transmission electron microscope (STEM) (JEOL Ltd., Akishima, Tokyo) with a voltage of 200 kV. The length and width of CNCs were assessed from the images using an Image J 1.53t software (Wayne Rasband and contributors, National Institutes of Health, Washington, DC, USA). The average particle size, polydispersity index, and zeta potential of diluted CNC sample (0.1 wt%) were assessed with a Zetasizer Nano ZS instrument (Malvern Instruments Ltd., Worcestershire, UK). The material refractive index is 1.50 while the dispersant refractive index is 1.33. The rheological behavior of CNCs at varied concentrations was assessed using a Discovery HR-2 rheometer (TA instruments, Newcastle, DE, USA) fitted with a DIN Concentric Cylinder featuring bob and cup geometry (bob diameter = 28 mm, cup diameter = 30.4 mm) according to the method we established previously [15]. Samples were loaded and maintained at 37 °C for 5 min, followed by a flow ramp test at 37 °C spanning a 180-s duration (sampling interval of 1 s/pt) and a range of shear rate from 1 to 100 s^−1^. Viscosity readings versus shear rate were recorded. Three tests were performed for each sample.

### 2.3. Preparation and Characterization of CNC-Stabilized Pickering Emulsion

CNC dispersion with varied concentrations was introduced into lemongrass essential oil (EO) while employing magnetic stirring; subsequently, ultrasonication was performed using a 500 W ultrasonicator at 60% amplitude (5 s on/2 s off) for 5 min. The emulsion comprised 5 wt% EO and specific concentrations of CNCs (0, 0.5 and 1.0 wt%). The thermodynamic stability of emulsion samples was then tested via centrifugation test, freeze–thaw test, heating–cooling test and room temperature storage test. The behavior of sample phase separation (if any) was recorded after each test. Centrifugation test was performed by subjecting the emulsion samples to a centrifugal force of 10,000 rpm (Eppendorf 5418, Enfield, CT, USA) for a duration of 15 min at 25 °C. The freeze–thaw tests comprised two cycles, with each cycle involving storage at −20 °C for 48 h followed by 25 °C for 48 h. Samples under heating–cooling test were subjected to six cycles of sample storage at 4 °C for 24 h followed by 45 °C for 24 h. Additionally, emulsion samples were stored at room temperature (shielded from light) and monitored for any potential phase separation over time.

Droplet size of CNC-stabilized Pickering emulsions was measured by using an optical microscope (50W Halogen Trinocular Microscope, AmScope, United Scope LLC, Irvine, CA, USA) right after the preparation of the sample. Specifically, an undiluted sample of 1 µL was applied to a slide, covered with a coverslip, and images were captured under a 40× objective magnification. The determination of emulsion droplet size was performed using an Image J 1.53t software (Wayne Rasband and contributors, National Institutes of Health, Washington, DC, USA). 

### 2.4. Effect of NaCl on CNC and CNC-Stabilized Pickering Emulsion

In order to determine the effect of NaCl on CNCs, CNC suspension (1 wt%) in the presence of various concentrations of NaCl (1 mM, 10 mM, and 40 mM) (i.e., salted CNC) was prepared and characterized. Specifically, CNCs and NaCl were mixed and sonicated as shown in Section 2.3. Particle size and zeta potential of salted CNCs were determined by the zetasizer to reflect the effect of NaCl on hydrodiameter and surface charge of CNCs. All experiments were conducted in triplicate at 23 °C. The refractive indices of the material and dispersant are 1.50 and 1.33, respectively. Before measurement, samples were diluted with deionized water at a ratio of 1:100. Viscosity of salted CNCs was also determined with the similar method as described above but at 23 °C.

Emulsions were formulated with 5% EO, 1 wt% CNC, and varied concentrations of NaCl (1 mM, 10 mM, and 40 mM). The emulsion was prepared via sonication (using the same parameters as above) to obtain Pickering emulsion. The stability of salted CNC-stabilized Pickering emulsion was also tested as described above. Particle size and zeta potential of salted CNC-stabilized Pickering emulsion were determined by utilizing the Zetasizer. The samples underwent dilution with water, following the procedures outlined earlier. 

The stability of emulsion against creaming was assessed through the creaming index using the formula below.

(3) $$Creaming\;index=\frac{V_{S}}{V_{T}}\times 100\%$$

where $V_{S}$ represents the volume of the serum layer and $V_{T}$ represents the overall volume of the emulsion sample.

Encapsulation efficiency of the emulsion sample after centrifugation was also determined by the equation below [16].

(4) $$Encapsulation\;efficiency=\frac{V_{E}}{V_{T}}$$

where $V_{E}$ represents the fraction of volume occupied by the encapsulated emulsion, and $V_{T}$ represents the overall volume of the emulsion sample. 

### 2.5. Preparation and Characterization of Emulsions Stabilized with CNC and Tween 80

Emulsion samples containing both CNC and a low molecular weight surfactant (namely, Tween 80) were also formulated. The emulsions loaded with essential oil comprised 5 wt% lemongrass EO, 10 wt% Tween 80 and varying concentrations of CNCs (0, 0.25, 0.5, and 1 wt%). Emulsions were created by gradually introducing Tween 80 to EO with stirring. Subsequently, the water phase containing CNCs was added while stirring, following that, ultrasonication was applied using the same procedures as above. Thermodynamic stability, particle size and zeta potential of the emulsions were tested as above. 

Emulsion samples were also prepared with salted CNCs and Tween 80. The emulsions contained 5 wt% EO, 10 wt% Tween 80, 1 wt% CNC and varying concentrations of NaCl (0, 1, 10, and 40 mM). The size of particles, zeta potential and thermodynamic stability of the emulsions were also assessed. 

### 2.6. Statistical Analysis

All experiments were conducted with at least triplicate measurements. Statistical significance (α = 0.05) was evaluated using Duncan’s tests with SAS/STAT software (SAS Institute Inc., Cary, NC, USA). Samples labeled with distinct letters exhibited significant differences (Duncan, *p* < 0.05) when compared to each other.

## 3. Results and Discussion

### 3.1. Yield and Color of Soybean Stover Following Each Treatment

Table 1 shows the yield and color parameter of soybean stover following each treatment. Specifically, raw, washed and alkaline treated soybean stover show brown color while the bleached sample appeared whitish due to the removal of lignin (as shown by an increased L* value but decreased a* and b* values). After acid hydrolysis, the soybean CNC suspension showed a slight yellowness (corresponding to a decreased L* value but slightly increased a* and b* values), probably due to the treatment of fiber by sulfuric acid. 

The reported composition of chemicals in soybean stover includes ~42% cellulose, ~17% hemicellulose, ~22% lignin and 0.2% ash [4]. Table 1 indicates a decrease in overall yields following each treatment. Similar to our previous results on corn stover [15], each treatment step causes some reduction in the overall yield. The washing process had a resulting yield of ~75%. The reduction in the course of the washing procedure is likely attributed to the elimination of ash, water-soluble extractives and small particles with a size below 55 µm. Alkaline treatment was performed to solubilize pectin, hemicellulose, lignin and proteins while bleaching treatment was mainly used to remove lignin residuals [17]. The overall yield of soybean stover after washing, alkaline and bleaching treatment in this study was ~29%, which was lower than the yield of soy hull after alkaline and bleaching treatment (46%) [17]. The difference in the yields may be due to the difference in the chemical composition of the soybean stover used in this study and soybean hull, as well as differences in treatment conditions. 

The step yield of soybean CNCs from bleached soybean stover is around 17 wt%. This yield is similar to that reported for maize CNCs (15.6%) [18], soy hull CNCs (8~20 wt%) [17] and CNCs produced from corncob via sulfuric acid hydrolysis [19]. The loss of samples during acid hydrolysis treatments was probably due to the hydrolysis of amorphous regions of cellulose. The overall yield of soybean CNCs prepared from raw soybean stover is around 5 wt%, which shows that soybean CNCs can be abundantly produced from soybean stover. The further enhancement in the overall yield can be improved via using smaller sieves during washing steps and probably by optimizing the parameters in each treatment. 

### 3.2. Characterization of Soybean CNC

**Figure 1:**
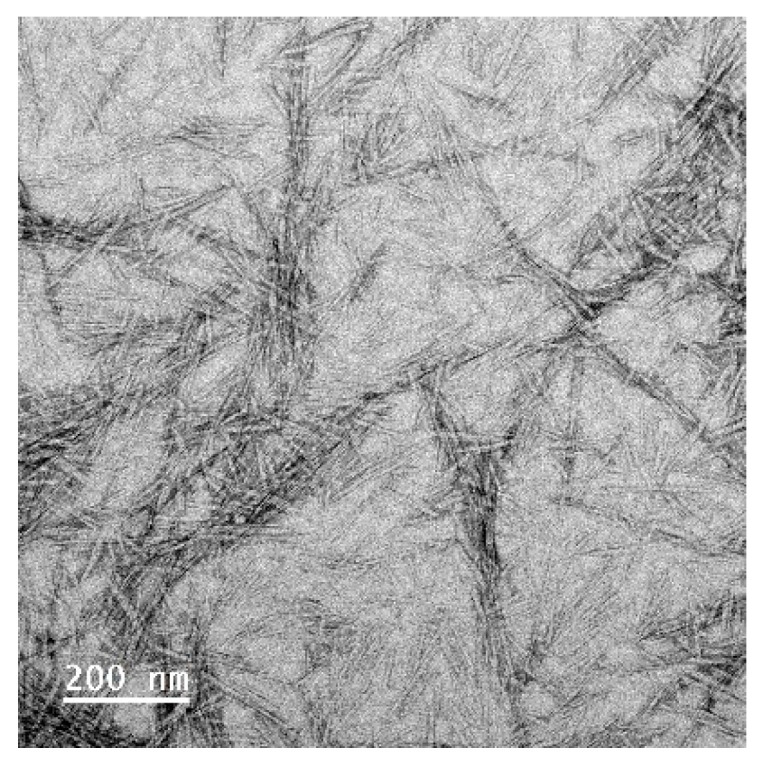
TEM image of soybean-stover-derived CNCs.

TEM image (Figure 1) shows that CNCs derived from soybean stover had a length of 117 ± 40 nm and width of 7.3 ± 2.0 nm. The average particle size, polydispersity index and zeta potential of soybean CNCs as measured by the Zetasizer was found to be 197 ± 6 nm, 0.65 and −50 ± 1 mV. The particle size result measured by the Zetasizer is slightly larger than that by TEM as the former measures the hydrodynamic diameter of CNCs. The particle size and zeta potential of soybean CNCs in this study closely align with values previously reported by other researchers [20,21,22]. Typically, the length of CNCs vary from 100~250 nm up to several microns depending on the sources, and the width varies from 5 nm to 70 nm [3]. For instance, the length and width of CNCs prepared from soybean straw were 100~600 nm and 9.4 nm, respectively, based on TEM images [6]. The particle size of CNC can vary, which is attributed to disparities in the sources of cellulose materials and variations in preparation conditions [21]. The zeta potential of CNCs generated through sulfuric acid hydrolysis may fall within the range of −11 to −52 mV, contingent upon the specific preparation techniques employed and the salinity of the dispersion [21,22,23].

**Figure 2:**
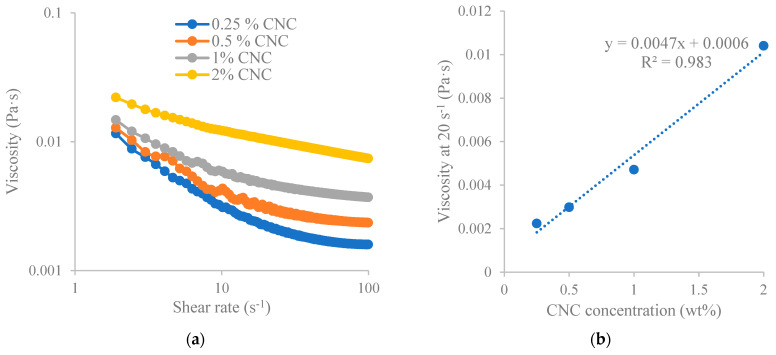
Viscosity characteristics of soybean-stover-derived CNCs. (**a**) Viscosity in relation to shear rates; (**b**) viscosity in relation to CNC concentration at a chosen shear rate (20 s^−1^).

The viscosity of soybean CNCs at varied concentrations against the shear rate was depicted in Figure 2a, exhibiting shear-thinning characteristics. In addition, a linear relationship was found between shear viscosity at 20 s^−1^ shear rate and the CNC concentration (Figure 2b). Higher CNC concentrations resulted in higher shear viscosity. Shear thinning behavior of nanocellulose was also observed in other studies [24,25]. The relationship between viscosity and nanocellulose concentration depends on the nanocellulose particle size (or aspect ratio), surface chemistry and surface charge [21]. In our prior investigation [15], it was demonstrated that the most suitable fit for the relationship between shear viscosity at a shear rate of 20 s⁻^1^ (η) and the concentration (C) of carboxylated nanocellulose was achieved using a power law model (log_10_ η = a + blog_10_ C). Similarly, wood-derived CNC (produced by USDA’s Forest Products Laboratory) also showed a Power law relationship between shear viscosity at 20 s^−1^ shear rate and the CNC concentration (0.23~2.33%) [25]. The differences in the fitting relationship between soybean CNCs (this study) and wood-derived CNCs [25] may be due to the differences in CNC preparation procedures and material sources. The relationship between viscosity and polymer concentration was also reported for other polymers such as hydroxyethyl cellulose [26].

### 3.3. CNC-Stabilized Pickering Emulsion

**Figure 3:**
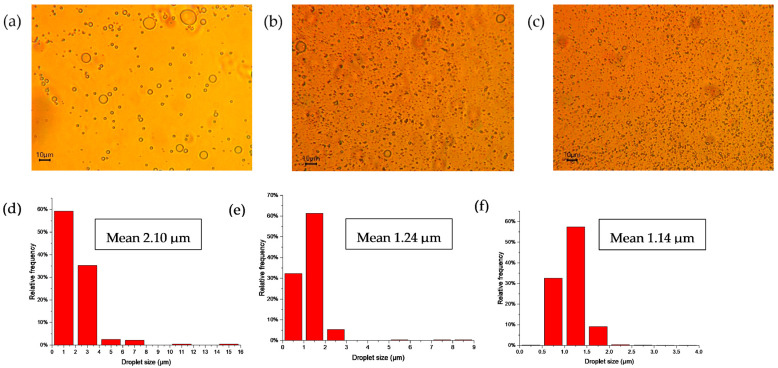
Optical microscopic images (**a**–**c**) and size distribution of droplets (**d**–**f**) in emulsions loaded with lemongrass essential oil (5%), stabilized with soybean CNCs at varied concentrations. (**a**,**d**) 0 wt% CNC; (**b**,**e**) 0.5 wt% CNC; and (**c**,**f**) 1.0 wt% CNC. Note: All the scale bars are 10 µm.

According to Figure 3a–c, the emulsion droplet size decreased at an increasing CNC concentration from 0% to 1%. Specifically, according to Figure 3d–f, the mean droplet size of emulsions decreased from 2.10 µm (at 0% CNC) to 1.14 µm (at 1.0% CNC). The effect of the biopolymer concentration on the droplet size of the Pickering emulsion has been reported [15,27]. In general, the Pickering emulsion stabilized by higher concentrations of biopolymer had better emulsion stability against creaming, and the emulsion droplet size was smaller compared to emulsions stabilized by lower concentrations of biopolymers. In the case of the CNC-stabilized Pickering emulsion, higher CNC concentrations resulted in more surface coverage of CNCs at the oil–water interface and therefore more emulsion droplets and a smaller emulsion droplet size [11]. A similar size range (1~4 µm) as measured by optical microscopy was reported for canola oil-in-water or hexadecane-in-water emulsions stabilized by CNCs [14].

Thermodynamic stability of the Pickering emulsion stabilized by soybean CNCs is shown in Table 2. Results from Table 2 reveal that the CNC-stabilized Pickering emulsion was not stable against all the thermodynamic stability tests. Phase separation was observed in all the stability tests as the Pickering emulsion creamed quickly. As shown in Figure 3b,c, the CNC-stabilized Pickering emulsion formed flocculation, though it had a smaller droplet size. Similarly, our previous study [15] showed that emulsions stabilized by corn-stover-derived TEMPO-CNF were effective against coalescence but not flocculation. Nanocellulosic materials could stabilize the emulsion against coalescence but not flocculation [28]. Aw, Lim, Low, Singh, Chan and Tey [11] also reported that no stable emulsion was obtained when using CNCs solely as the stabilizer. 

### 3.4. Effect of NaCl on CNC and CNC-Stabilized Pickering Emulsion

According to Table 3, the mean particle size of soybean CNCs increased upon the increase in NaCl concentration. Specifically, the particle size increased from 86 nm (0 mM NaCl) to 137 nm (40 mM NaCl). Different concentrations of NaCl did not affect the polydispersity index of the CNC suspension. Upon the increase in NaCl concentration from 0 mM to 40 mM, the zeta potential of the CNC suspension is less negative (changing from −45 mV to −39 mV). The hydrodynamic diameter of CNC particles increased in the presence of higher NaCl concentration due to CNC aggregation [29]. NaCl could cause CNC to form aggregation and gelation via screening the surface charges of CNC [30], and therefore, the absolute zeta potential of the CNC suspension reduced in the presence of NaCl. The effect of NaCl on the viscosity profile of soybean CNC was shown in Figure S1 (Supplementary Material). As shown in Table 3, the increase in NaCl concentration resulted in higher CNC viscosity. Specifically, with the presence of 40 mM NaCl, the shear viscosity of CNCs increased significantly (0.216 Pa·s) as compared to the viscosity at 0 mM NaCl (0.006 Pa·s). The increase in ionic strength was shown to compress the electric double layer, allowing CNC strands to interact with each other, and cause gelation in CNCs, thus resulting in higher viscosity [31]. 

In order to evaluate the interfacial stabilization capability of CNCs with the presence of NaCl, the emulsion stability, particle size and zeta potential were determined. Results from Table 4 showed that the presence of NaCl slightly increased the mean particle size of the emulsion, with no significant differences towards the zeta potential of emulsions. The variance in the mean particle size observed in the emulsion with 0 mM NaCl, as determined by optical microscopy (1.14 μm as depicted in Figure 3f), and that determined by Zetasizer (utilizing dynamic light scattering, 393 nm as shown in Table 4) could be attributed to several factors. Firstly, optical microscopy is limited by a detection threshold, unable to visualize substances below 0.2 μm, whereas the Zetasizer offers a wider detection range spanning from 0.3 nm to 10 μm. Secondly, while the raw emulsion was employed for optical microscopy imaging, the emulsion underwent a dilution with water (at a ratio of 1:100) for Zetasizer measurements. This dilution with water has the potential to impact the emulsion droplet size.

According to Table 5, with the presence of 40 mM NaCl, the emulsions were still not stable against centrifugal forces and heating–cooling cycles, but the emulsions did not form phase separation after freeze–thaw cycles and room temperature storage for at least 30 days. Even though the emulsion stabilized by salted CNCs still formed phase separation after centrifugation, the emulsion phase increased as reflected by the change in the creaming index and encapsulation efficiency. Specifically, results from Table 6 showed that the presence of 40 mM NaCl enhanced the encapsulation efficiency of EO from <5% to 78% and reduced the creaming index from 91% to 0%. A similar phenomenon has been reported by Varanasi et al. [14]. Specifically, the addition of salt led to CNC aggregation and increased the emulsion volume. The addition of salt was also shown to minimize the quantity of CNCs needed for stable emulsion formation [14]. Similarly, according to Aw et al. [11], the addition of NaCl aided in the formation of CNC-stabilized Pickering emulsion, and the encapsulation efficiency of curcumin in the salted CNC (i.e., in the presence of 40 mM NaCl) stabilized Pickering emulsion was nearly 100%. Salted CNC was shown to act as a barrier both physically and chemically at the interface between oil and water, inhibiting curcumin degradation in CNC-stabilized Pickering emulsions loaded with curcumin [11]. Besides CNCs, NaCl was also shown to enhance the emulsion stability of other polysaccharide-stabilized emulsions [32]. For instance, research demonstrated that the addition of NaCl improved the storage stability of emulsions stabilized with carboxymethyl starch and xanthan gum [32]. 

The emulsion stabilized by salted CNCs (in the presence of 40 mM NaCl) did not form phase separation after freeze–thaw cycles, indicating that the presence of NaCl enhanced the stability of the CNC-stabilized emulsion against freeze–thaw cycles. Salted CNCs (i.e., 0.5 wt% CNC in 10 mM NaCl) were shown to inhibit ice recrystallization [33]. The ice recrystallization inhibition activity displayed by salted CNCs could reduce ice formation during freeze–thaw process and its influence on emulsion stability. In addition, salted CNCs could form a thick interfacial layer around oil droplets, which protects against crystal penetration and thus improve the emulsion stability during freeze–thaw process [15].

The impact of NaCl on the CNC-stabilized emulsion was probably due to two factors: (1) the charge screening effect of NaCl caused CNC aggregates which were reported to have higher adsorption energy at the oil–water interface than unsalted CNC [14]; (2) CNC aggregates had higher viscosity which contributed to enhanced emulsion stability. The adsorption of Na^+^ to the CNC surface shielded the surface charge of CNCs, diminishing electrostatic repulsion among CNC molecules. Consequently, more CNCs could adsorb onto the interface between oil and water, leading to a more stable emulsion [14]. Similarly, salt was also reported to influence the rheology of negatively charged cellulose nanofibrils, and it also reduced the cellulose nanofibril concentration needed to maintain the stability of oil-in-water emulsions [34]. The surface charge of CNCs was reported to have an impact on the emulsion stability [35]. Kalashnikova, Bizot, Cathala and Capron [35] reported that no emulsion was observed when using sulphated cotton cellulose nanocrystals to prepare the Pickering emulsion, and in order to prepare the Pickering emulsion, the surface charge density of sulfated CNCs should be less than 0.033 e/nm^2^. The charge density of sulfated CNCs could be adjusted by the addition of mild HCl or salts [14]. 

### 3.5. Nanoemulsions Stabilized by CNCs and Tween 80

Emulsions stabilized by CNCs (or salted CNC) and Tween 80 were also studied. As shown in Table 7, the mean particle size of the Tween 80-stabilized emulsion is 49 nm, but the emulsion was not colloidally stable with a zeta potential of ~−10 mV. With the presence of CNCs or salted CNCs, the mean particle size increased slightly but was still within nanometer range (~70 nm). The particle size of the nanoemulsion formulated by Tween 80 and salted CNCs in this study was much smaller compared to the Pickering nanoemulsion stabilized by particles and surfactant blends which mostly have a size larger than 150 nm [36]. For instance, cyclodextrin–Tween 20-stabilized Pickering nanoemulsion had a size of around 200 nm [37]. Nanoemulsion stabilized by sodium caseinate, Tween 20 and beta-cyclodextrin had a mean droplet size of 155 nm [38]. In addition, the emulsion stabilized by Tween 80 and salted CNCs in this study was more colloidally stable (zeta potential of ~−37 mV) as compared to the emulsion stabilized by Tween 80 and unsalted CNCs (−22 mV). This indicates the good colloidal stability of the nanoemulsion stabilized by Tween 80 and salted CNCs.

Thermodynamic stability tests were conducted on the nanoemulsion, and comparisons were drawn among emulsions stabilized with Tween 80, Tween 80 + CNC and Tween 80 + salted CNC. Table 8 shows that all the formulated nanoemulsions with or without CNCs/salted CNCs were physically stable against centrifugal forces (corresponding to 100% encapsulation efficiency) and after room temperature storage for at least 30 days. However, the Tween 80-stabilized emulsion was not stable against freeze–thaw and heating–cooling processes. The explanation for the stability of the Tween 80-stabilized emulsion against thermodynamic tests was discussed in our previous study [15]. Similarly, the emulsion stabilized by Tween 80 and CNCs (0.25 wt%~1 wt%) formed phase separation after freeze–thaw cycles and heating–cooling cycles, but the emulsion stabilized by Tween 80 and salted CNCs (10~40 mM NaCl) did not form phase separation after freeze–thaw and heating–cooling cycles. This indicates that at certain NaCl concentrations, salted CNCs can enhance the stability of emulsions stabilized with Tween 80 during freeze–thaw and heating–cooling cycles. Similarly, our previous study [15] showed that TEMPO-CNF could enhance the stability of Tween 80-stabilized emulsions during freeze–thaw cycles. It is postulated that similar mechanisms exist for salted CNCs in improving the freeze–thaw stability of Tween 80-stabilized emulsions. As discussed in Section 3.4, salted CNCs possessed ice recrystallization inhibition activity and could form a thick interfacial membrane around oil droplets, thus it is effective as an emulsion stabilizer against freeze–thaw cycles. 

The binary emulsifier system (e.g., Tween 80 and salted CNC) was effective against emulsion destabilization during the heating–cooling process, but the emulsion stabilized by salted CNCs (Table 5) or Tween 80 was not. Therefore, there existed synergistic effects between salted CNCs and Tween 80 in the emulsion stability against heating–cooling cycles. The variation in temperature during heating–cooling influences the dynamics of surfactant molecules and the surface tension of water. Emulsions stabilized by thin films (e.g., emulsions stabilized by Tween 80) have a higher coalescence frequency at an elevated temperature [39]. It is postulated that the thick interfacial layer formed by salted CNCs inhibited emulsion coalescence at increasing temperatures. To our knowledge, this study represents the initial reporting of the capability of salted CNCs in enhancing freeze–thaw and heating–cooling stability of emulsions stabilized by surfactants (e.g., Tween 80). 

Comparing the emulsion stability results of salted CNCs (as shown in Table 5) and salted CNC + Tween 80 (as shown in Table 8), it is observed that synergistic effects existed between Tween 80 and salted CNCs in the stabilization of emulsions against environmental stresses. Emulsions formulated by Tween 80 and salted CNCs were physically stable against all thermodynamic stability tests, while emulsions formulated by Tween 80, or salted CNCs, or Tween 80 + unsalted CNC formed phase separation after certain thermodynamic stability tests. Similarly, synergistic effects have been reported between pectin and Tween 80 [40] and between nanocellulose and saponin [41], as well as between nanocellulose and methyl cellulose [42] on emulsion stabilization. The synergism between salted CNCs and Tween 80 is meaningful to develop stable emulsion products against environmental stresses for various types of applications.

## 4. Conclusions

In this study, soybean stover-derived CNCs were prepared and studied as emulsion stabilizers. Soybean CNCs exhibited an average length and width of 117 nm and 7 nm, respectively, along with a zeta potential of −50 mV, suggesting colloidal stability. Soybean CNCs at concentrations of no less than 0.5 wt% stabilized the lemongrass essential oil-loaded oil-in-water emulsion against coalescence but not flocculation. The addition of NaCl (40 mM) enhanced the stability of the CNC-stabilized emulsion against freeze–thaw cycles. The presence of NaCl (40 mM) also increased the encapsulation efficiency of the CNC-stabilized emulsion to 78%. A stable nanoemulsion could be formulated with a mixed emulsifier system containing Tween 80 and salted CNCs (in the presence of 40 mM NaCl). The obtained nanoemulsion had a mean particle size of 71 nm and the nanoemulsion is colloidally stable with a zeta potential of −37 mV. The nanoemulsion stabilized by Tween 80 and salted CNCs was also stable against centrifugal forces, heating–cooling and freeze–thaw cycles, as well as room temperature storage for at least 30 days. This study highlighted the importance of salt in the formulation of CNC-stabilized Pickering emulsions. Synergistic effects existed between Tween 80 and salted CNCs in stabilizing oil-in-water emulsions. As far as we are aware, this study represents the first instance of reporting that salted CNCs can enhance the stability of Tween 80-stabilized emulsions during freeze–thaw cycles and heating–cooling cycles. Results obtained from this study demonstrate the feasibility of combining agricultural byproduct-derived nanocellulose and surfactants in the formulation of emulsion systems that are stable against environmental stresses. 

## Figures and Tables

**Table 1:** Yield and color of the soybean stover sample following each treatment step.

	Raw Sample	Washed	Alkaline Treated	Bleached	Acid Hydrolysis Treated
Step yield (%)	N/A	74.6 ± 4.3	58.6 ± 1.7	65.2 ± 2.0	17.3 ± 2.1
Overall yield (%)	N/A	74.6 ± 4.3	43.7 ± 2.8	28.5 ± 1.9	4.9 ± 0.7
L*	30 ± 3	37 ± 6	32 ± 4	49 ± 7	34.4 ± 2.5
a*	4.3 ± 2.2	4.3 ± 0.5	6 ± 2	0.5 ± 0.1	1.30 ± 0.01
b*	12 ± 1	12.3 ± 2.2	14.7 ± 0.9	4.9 ± 0.5	5.3 ± 0.3

**Table 2:** Thermodynamic stability of Pickering emulsions stabilized by soybean CNCs.

Lemongrass Essential Oil (wt%)	CNC (wt%)	After Centrifugation	After 2 Freeze–Thaw Cycles	After 6 Heating–Cooling Cycles	After 30 Days Room Temperature Storage
5	0	PS ^1^	PS	PS	PS
5	0.5	PS	PS	PS	PS
5	1	PS	PS	PS	PS

^1^ PS corresponds to phase separation.

**Table 3:** Effect of NaCl on particle size, zeta potential and shear viscosity of soybean CNC.

CNC (wt%)	NaCl (mM)	Mean Particle Size (nm)	Polydispersity Index	Zeta Potential (mV)	Viscosity under a Shear Rate of 20 s^−1^ (Pa·s)
1	0	86 ± 2 ^1a^	0.38 ± 0.01 ^a^	−45 ± 3 ^a^	0.006
1	1	86 ± 16 ^ab^	0.4 ± 0.1 ^a^	−36.6 ± 8.1 ^b^	ND ^2^
1	10	123 ± 26 ^b^	0.5 ± 0.3 ^a^	−36.4 ± 10.3 ^a^	ND
1	40	137 ± 11 ^ab^	0.4 ± 0.1 ^a^	−39.2 ± 3.0 ^ab^	0.216

^1^ The mean particle size of CNCs at 0 mM NaCl is smaller than that of CNCs in Section 3.2 as the former went through ultrasonication treatment. ^2^ Not determined. ^ab^ Samples labeled with distinct letters showed significant differences (Duncan, *p* < 0.05) when compared across various NaCl concentrations.

**Table 4:** Influence of NaCl on the particle size and zeta potential of emulsions stabilized with soybean CNCs.

Lemongrass Essential Oil (wt%)	CNC (wt%)	NaCl (mM)	Mean Particle Size (nm)	Polydispersity Index	Zeta Potential (mV)
5	1	0	393 ± 39 ^a^	0.43 ± 0.09 ^a^	−51 ± 4 ^a^
5	1	1	419 ± 15 ^a^	0.42 ± 0.06 ^a^	−39 ± 2 ^a^
5	1	10	547 ± 41 ^a^	0.5 ± 0.1 ^b^	−51 ± 2 ^a^
5	1	40	516 ± 36 ^a^	0.44 ± 0.07 ^a^	−46 ± 2 ^a^

^ab^ Samples labeled with distinct letters showed significant differences (Duncan, *p* < 0.05) when compared across various NaCl concentrations.

**Table 5:** Influence of NaCl on the thermodynamic stability of emulsions with stabilization provided by soybean CNCs.

Lemongrass Essential Oil (wt%)	CNC (wt%)	NaCl (mM)	After Centrifugation	After 2 Freeze–thaw Cycles	After 6 Heating–Cooling Cycles	After 30 Days Room Temperature Storage
5	1	0	PS ^1^	PS	PS	PS
5	1	1	PS	PS	PS	PS
5	1	10	PS	PS	PS	PS
5	1	40	PS	NPS	PS	NPS

^1^ PS corresponds to phase separation, NPS corresponds to no phase separation.

**Table 6:** Effect of NaCl on the creaming index of CNC-stabilized emulsions after centrifugation.

Lemongrass Essential Oil (wt%)	CNC (wt%)	NaCl (mM)	Creaming Index	Encapsulation Efficiency
5	1	0	91%	ND ^1^
5	1	1	90%	ND
5	1	10	90%	ND
5	1	40	0%	78%

^1^ ND refers to not determined, as it is less than 5%.

**Table 7:** Size of particles and zeta potential in emulsions stabilized with Tween 80 and CNCs.

Lemongrass Essential Oil (wt%)	Tween 80 (wt%)	CNC (wt%)	NaCl (mM)	Mean Particle Size (nm)	Polydispersity Index	Zeta Potential (mV)
5	10	0	0	49 ± 21 ^a^	0.3 ± 0.2 ^a^	−10 ± 2 ^a^
5	10	1	0	76 ± 25 ^a^	0.4 ± 0.3 ^a^	−22 ± 5 ^b^
5	10	1	40	71 ± 1 ^a^	0.63 ± 0.01 ^a^	−36.8 ± 0.4 ^c^

^abc^ Samples labeled with distinct letters exhibited significant differences (Duncan, *p* < 0.05) when compared to each other.

**Table 8:** Thermodynamic stability of emulsions stabilized by Tween 80 and CNCs.

Lemongrass Essential Oil (wt%)	Tween 80 (wt%)	CNC (wt%)	NaCl (mM)	After Centrifugation	After 2 Freeze–Thaw Cycles	After 6 Heating–Cooling Cycles	After 30 Days Room Temperature Storage
5	10	0	0	NPS ^1^	PS	PS	NPS
5	10	0.25	0	NPS	PS	PS	NPS
5	10	0.5	0	NPS	PS	PS	NPS
5	10	1	0	NPS	PS	PS	NPS
5	10	1	1	NPS	PS	PS	NPS
5	10	1	10	NPS	NPS	PS	NPS
5	10	1	40	NPS	NPS	NPS	NPS

^1^ NPS corresponds to no phase separation, and therefore, the emulsion encapsulation efficiency is 100%.

## Data Availability

Data and materials will be available upon request.

## Supplementary Materials

The following supporting information can be downloaded at: https://www.mdpi.com/article/10.3390/polym15244682/s1, Figure S1: Effect of NaCl on the viscosity profile of 1 wt% soybean CNC.
