# Supplementary figures and images for: Synergistic Stabilization of Nanoemulsion Using Nonionic Surfactants and Salt-Sensitive Cellulose Nanocrystals
Source: Polymers (Basel). 2023 Dec 12;15(24):4682. doi: 10.3390/polym15244682 (PMC10747914; doi:10.3390/polym15244682)

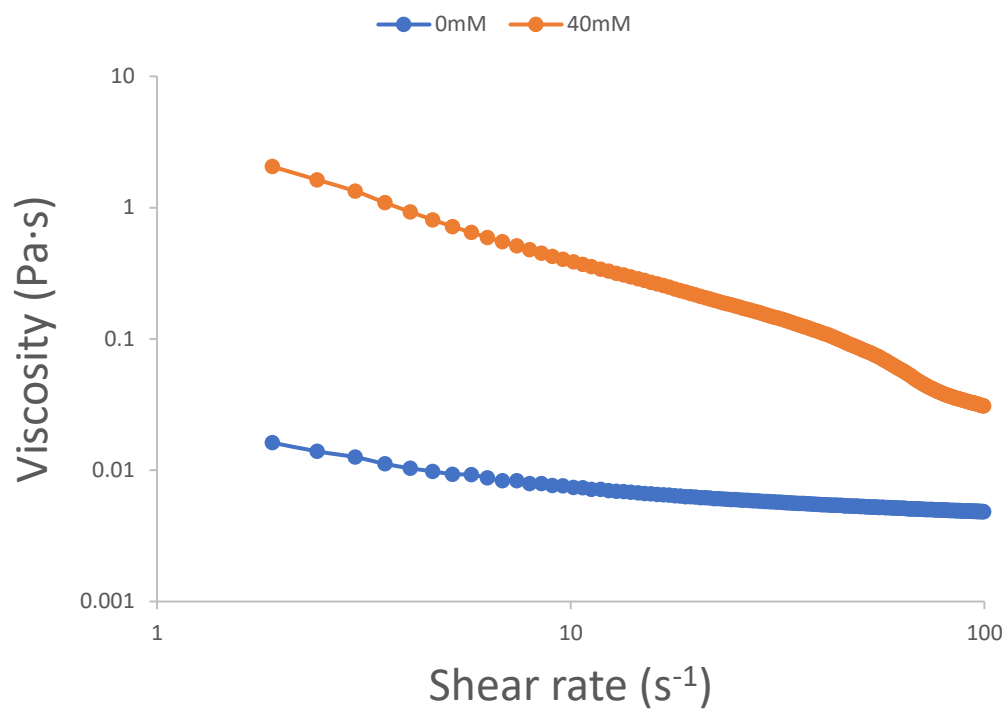

Figure S1 Effect of NaCl on the viscosity profile of 1 wt% soybean CNC

Supplement: Supplementary file 1 [file polymers-15-04682-s001.zip › polymers-2744359-supplementary.pdf]
